# Insulin-like peptide 5 (INSL5) positively correlates with anti-Müllerian hormone (AMH) in women with the polycystic ovary syndrome: a case-control study

**DOI:** 10.1186/s13048-022-01052-7

**Published:** 2022-10-28

**Authors:** Yijie Chen, Miao Deng, Zhaojing Chen, Shuyang Han, Jun Chen, Hongyan Zhang, Qianwen Wang, Xuejing Jin, Wenhua Liu, Zhifen Zhang

**Affiliations:** 1 The Fourth School of Clinical Medicine, Hangzhou Women’s Hospital, Affiliated Hospital of Zhejiang Chinese Medical University, Zhejiang, 310008 Hangzhou China; 2 Department of the Reproductive Endocrinology Division, Hangzhou Women’s Hospital (Hangzhou Maternity and Child Health Care Hospital), No. 369 kunpeng Road, Shangcheng District, Hangzhou, 310008 Zhejiang China; 3 Department of Epidemiology and Health Statistics, Hangzhou Normal University School of Public Health, Hangzhou, 311121 China; 4 Department of Obstetrics and Gynecology, Nanjing Medical University, Nanjing, 210000 China; 5 Department of fourth Clinical Medical College, Zhejiang Chinese Medical University, 548 Binwen Road, Binjiang District, Hangzhou, 310053 Zhejiang China

**Keywords:** Insulin-like peptide 5, Anti-Müllerian hormone, Polycystic ovary syndrome

## Abstract

**Background:**

Insulin-like peptide 5 (INSL5) is involved in both reproductive and metabolic processes in polycystic ovary syndrome (PCOS). This study aimed to evaluate the relationship between INSL5 and anti-Müllerian hormone (AMH).

**Methods:**

A retrospective case–control study was conducted in a university-based reproductive centre between December 2019 and January 2021. We included 117 women with PCOS and 100 healthy subjects from Zhejiang Province. All subjects were divided into four groups (1st–4th) based on quartiles of serum INSL5 levels. Serum INSL5 concentration was assayed using an enzyme-linked immunosorbent assay.

**Results:**

A significant direct association was observed between serum INSL5 and AMH levels in women with PCOS. The mean AMH level in the 1st–4th INSL5 level quartiles were 4.64, 5.20, 6.46, and 9.48 ng/ml, respectively (*P* < 0.001). After adjusting for age, body mass index, metabolic indices, and serum levels of oestradiol and total testosterone, AMH levels remained positively and significantly associated with INSL5 levels (*P* for trend < 0.001). The diagnostic value of AMH was better than that of INSL5.

**Conclusions:**

INSL5 and AMH levels were significantly correlated and elevated in women with PCOS. INSL5 and AMH might be associated with increased androgen secretion and chronic anovulation in PCOS.

## Introduction

Polycystic ovary syndrome (PCOS) is both a reproductive and metabolic disorder in women. PCOS has a wide variety of clinical presentations and symptoms, including hyperandrogenism, oligo or amenorrhoea and polycystic ovaries (PCO) [1, 2]. The estimated prevalence of PCOS is 8-13% [3]. Familial clustering studies have shown that PCOS has a strong heritable component [4]. Characteristics of the condition are heterogeneous. PCOS features can include cystic ovaries, metabolic disorders, reproductive and physiological abnormalities, and higher risk of cardiovascular disorders [5, 6]. PCOS is also characterised by abnormal follicular development, with the number of growing follicles twice that in typical preovulatory arrest [7].

One of the important biomarkers used to diagnose PCOS is anti-Müllerian hormone (AMH), which is a polypeptide of the transforming growth factor beta family [8]. AMH plays an important role in sexual differentiation in male foetuses and is solely secreted by the granulosa cells of the pre-antral and small antral ovarian follicles in women [9, 10]. AMH inhibits the recruitment of primordial follicles from the resting oocyte pool, which may inhibit the role of follicle-stimulating hormone (FSH), resulting in female ovulation disorders [11]. The changes in AMH precedes modifications in serum FSH, inhibin B, and antral follicle count. AMH secretion drops significantly before menopause due to follicular failure [12]. The levels of AMH in patients with PCOS are significantly correlated with the number of ovarian small follicles, which reflects the presence of an ovarian follicle pool in the early follicular phase of the menstrual cycle and is strongly dependent on the number of antral follicles [13–15]. Tata et al. showed that AMH concentrations during pregnancy are two-to-three fold higher in women with PCOS as compared to women with no reproductive defects, and the severity of the reproductive dysfunction is positively correlated with AMH levels [11, 16]. Elevated AMH levels in PCOS were originally thought to be due only to an increase in the number of follicles [17, 18], as a low level of antral follicle count may lead to low serum AMH concentrations. Moreover, the level of serum AMH does not depend on the menstrual cycle phase or exogenous sex steroids [19].

Relaxin peptides are members of a superfamily of peptides including relaxin-1, relaxin-2, insulin-like peptide 3 (INSL3), INSL4, and INSL5, which play a vital physiological role in various diseases [20, 21]. Relaxin peptides interact with four G protein-coupled receptors (GPCRs), which are currently classified as relaxation protein family polypeptide receptors 1–4 (RXFP1–4). INSL5 is mainly expressed in the intestinal endocrine L-cells of the gastrointestinal system, especially the colon and rectum; but also by various tissues such as hypothalamus, uterine and pituitary tissue [22]. The connection between the reproductive system and INSL5 has been observed in INSL5 knockout mice. Animal experiment suggest that knockout INSL5 gene results in a notable decrease in sperm motility and irregular cycle in both sex [23]. INSL3 and AMH levels are significantly associated and elevated in women with PCOS which may reflect the dysfunction of follicles and granulosa cells [24]. Some animal studies have shown that INSL3 plays a vital role in developing ovarian follicular, while impaired fertility is associated with prolonged oestrous cycles and accelerated follicular atresia [25–27]. In a cross-sectional study, serum INSL5 levels were significantly higher in women with PCOS compared to women without PCOS (27.63 ± 7.74 vs.19.90 ± 5.85 ng/ml, *P* < 0.001) [28]. Another study suggested that the expression and localisation of RXFP4 on human sperm and INSL5 may affect male reproductive function [29].

Both INSL5 and AMH are recognised for their importance to reproductive function. They have recently been detected in adulthood in both sexes, where their roles still need to be explored. With this background, we assessed serum levels of AMH and INSL5 in women with PCOS, and we evaluated the relationship of these biochemicals with hormone levels and metabolic parameters.

## Methods

### Study population

A retrospective case-control study design was used for this study. The trial process took place from December 2019 to January 2021 in the Department of Endocrinology, Hangzhou Women’s Hospital (Hangzhou Maternal and Child Health Hospital) in Zhejiang, China. The study sample included 117 women with PCOS and 100 women with normal menstrual cycles, with ages ranging from 18 to 35 years. A detailed medical assessment (such as height, weight, family history of PCOS, regularity and length of the menstrual cycle, and medical history, etc.) was obtained from all subjects at the initial examination. Written consent was obtained from all participants. This study was approved by the ethics committee of Hangzhou Women’s Hospital (2018-004-No. 03).

### Inclusion and exclusion criteria

The subjects in the PCOS group were selected based on the Rotterdam consensus criteria (2018) [1]. Women with other causes of irregular menstrual cycles and/or androgen excess such as hyperprolactinemia, congenital adrenal hyperplasia, Cushing’s syndrome, or other diseases of the galactorrhoea, breast feeding, pregnancy adrenal gland, and thyroid disorders were excluded by laboratory analysis and clinical examination. The use of medications for hormonal contraception and/or anti-androgen therapy within the preceding 6 months, dyslipidaemia, obesity, hypertension, hyperglycaemia and insulin resistance were also criteria for exclusion from this study. The subjects in control group were enrolled with normal menstrual cycle without concomitant health problems or signs of hirsutism, acne or hyperandrogenism. No obvious abnormality in ultrasound imaging.

### Laboratory evaluation and biochemical assays

Fasting peripheral venous blood samples (2–3 mL) for fasting glucose, fasting insulin, oestradiol (E2), luteinizing hormone (LH), FSH, prolactin (PRL), progesterone (P), high-density lipoprotein (HDL), low-density lipoprotein (LDL), dehydroepiandrosterone sulfate (DHEA-S), total testosterone (T), sex hormone-binding globulin (SHBG), and AMH were drawn between 07:30 and 09:30 a.m. after an overnight fast. Sex hormone assessments were performed during the early follicular phase of menstrual bleeding (3rd to 5th days) and serum DHEA-S, T, SHBG, AMH were detected using the chemiluminescence method (Beckman Coulter UniCel Dxl-800). The levels of fasting plasma glucose, HDL, and LDL were measured using a Beckman Coulter AU5821 chemistry analyser. After separation, the excess serum samples were stored in a refrigerator at − 80 °C prior to analysis of INSL5. The levels of serum INSL5 were detected using a commercially available human enzyme-linked immunosorbent assay kit (Catalog number: JL19996, Jianglai biological, Hangzhou, China). The free androgen index (FAI) was calculated by the following formula: [FAI = 100x(T/SHBG)]. Insulin resistance was estimated using the homeostatic model assessment index of insulin resistance: HOMA-IR = [fasting insulin (pmol/L) × fasting glucose (mmol/L)] / (22.5 × 6.965).

### Statistical methods

The distributions of AMH and INSL5 were both skewed. IBM-SPSS 21.0 statistics software (IBM Inc., Armonk, NY, USA) was used for statistical processing. Data are expressed as mean ± standard deviation (SD). Continuous variables were analysed using analysis of variance (ANOVA) for comparing the characteristics across quartiles of INSL5. Receiver operating characteristic (ROC) curve and area under the curve (AUC) were used to evaluate the performance of INSL5 and AMH in building a risk model to diagnose PCOS. The association between INSL5 and AMH was evaluated using a linear regression model. To evaluate the potential for confounding, six models were built. Model 1 was unadjusted. Model 2 included adjustments for age and body mass index (BMI). Model 3 included the further adjustments of glucose metabolism indexes (fasting glucose and fasting insulin). Model 4 included the adjustments of model 3 and lipid metabolism indexes (total cholesterol and triglyceride). Model 5 included the adjustments of model 4 and lipid metabolism indexes (HDL and LDL). Model 6 included the adjustments of model 5 and sex hormones (E2 and T). Linear trends across the quartiles of INSL5 levels were assessed by modelling the median values of all quartiles as a continuous variable. *P* value < 0.05 was accepted as statistically significance.

## Results

### Background characteristics of the study population

The background sample characteristics are shown in the Table 1. The participants were divided into four groups based on quartiles of serum INSL5 levels: 1st (3.92–16.47)，2nd (16.47–21.26), 3rd (21.26–30.02) and 4th (30.02–99.84) ng/ml. There was a significant correlation between BMI and INSL5 (*P* = 0.033). A significant inverse association was observed between INSL5 levels and age across the quartiles (*P* = 0.02) in Table 1.

**Table 1:**
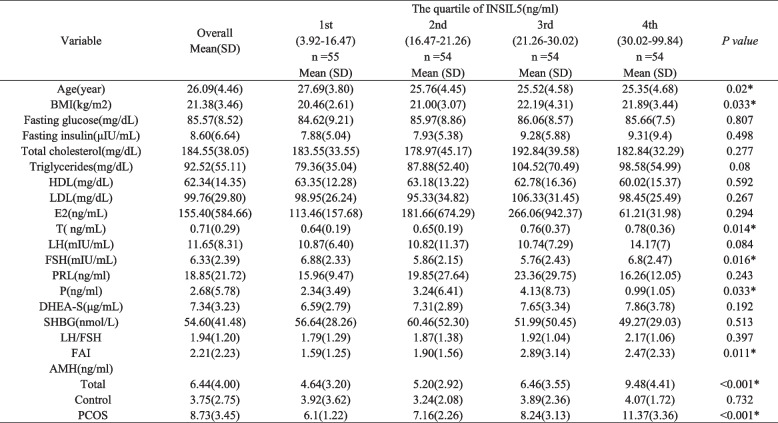
Background characteristics of the study population

### INSL5 and hormone levels

The hormone levels, lipid metabolism index and glucose metabolism index in the four groups are presented in Table 1. Significant proportional associations were observed between T levels and INSL5 across the quartiles. The mean T in 1st–4th were 0.71, 0.64, 0.76, and 0.78 ng/mL, respectively (*P* = 0.014). FAI, FSH and P also showed significant correlations with INSL5 across groups (*P* = 0.011; *P* = 0.016; *P* = 0.033, respectively). There were no significant differences among the groups in E2, LH, PRL, DHEA-S and SHBG (*P* > 0.05).

### INSL5 and AMH

**Fig. 1:**
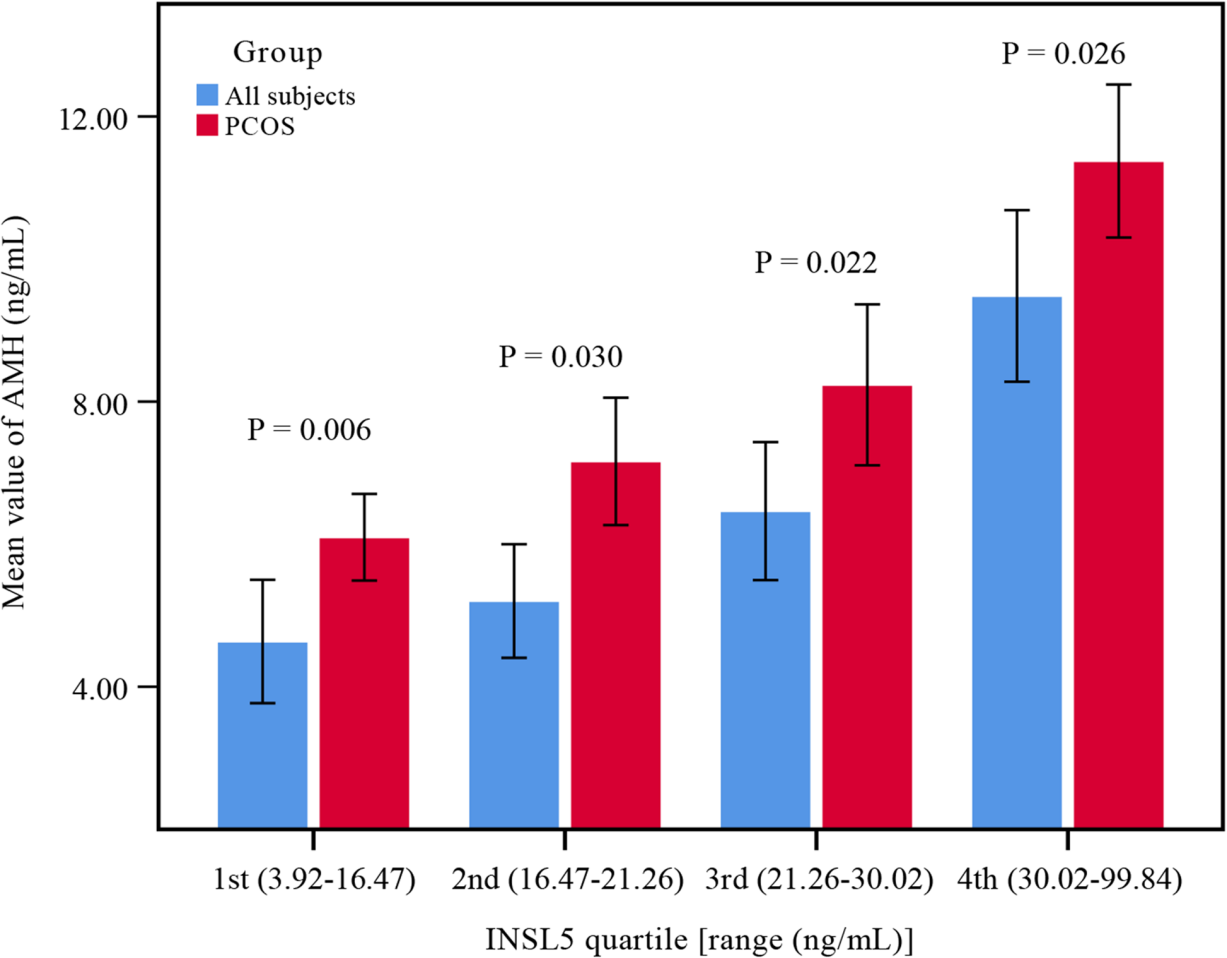
AMH level in INSIL5 quartiles. Standard deviations of the means are shown in the bars

In only-PCOS and the total groups, we found a positive correlation between INSL5 and AMH (*P* < 0.001). While there was no significant correlation found between INSL5 and AMH in the control group (*P* = 0.732), as shown in Table 1. Women with PCOS had higher serum AMH levels in the second, third and fourth INSL5 quartiles compared with the PCOS group and total groups (Fig. 1). Significant proportional relationships were observed between AMH levels and INSL5 across the quartiles in all subjects. Linear regression models were designed to reveal the independent relationship between INSL5 and AMH; The mean AMH in 1st–4th were 4.64, 5.20, 6.46, and 9.48, respectively (*P* < 0.001) in total subjects. The mean AMH in 1st–4th were 6.1, 7.16, 8.24 and 11.37 ng/mL, respectively (*P* < 0.001) in women with PCOS. In models 1 and model 2, the mean AMH increased with increasing INSL5 levels (Table 2). After further adjustments for glucose metabolism parameters in model 3, this association remained significant (*P* for trend < 0.001). Adjustment for lipid metabolism parameters had minimal effect on the results, with higher AMH levels remaining significantly associated with higher INSL5. There was a significant correlation between serum AMH and INSL5 in model 6 (*P* for trend < 0.001).

**Table 2:**
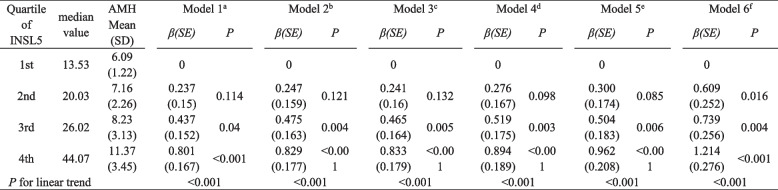
Association of serum INSIL5 and AMH among PCOS women

### INSL5 and PCOS

**Fig. 2:**
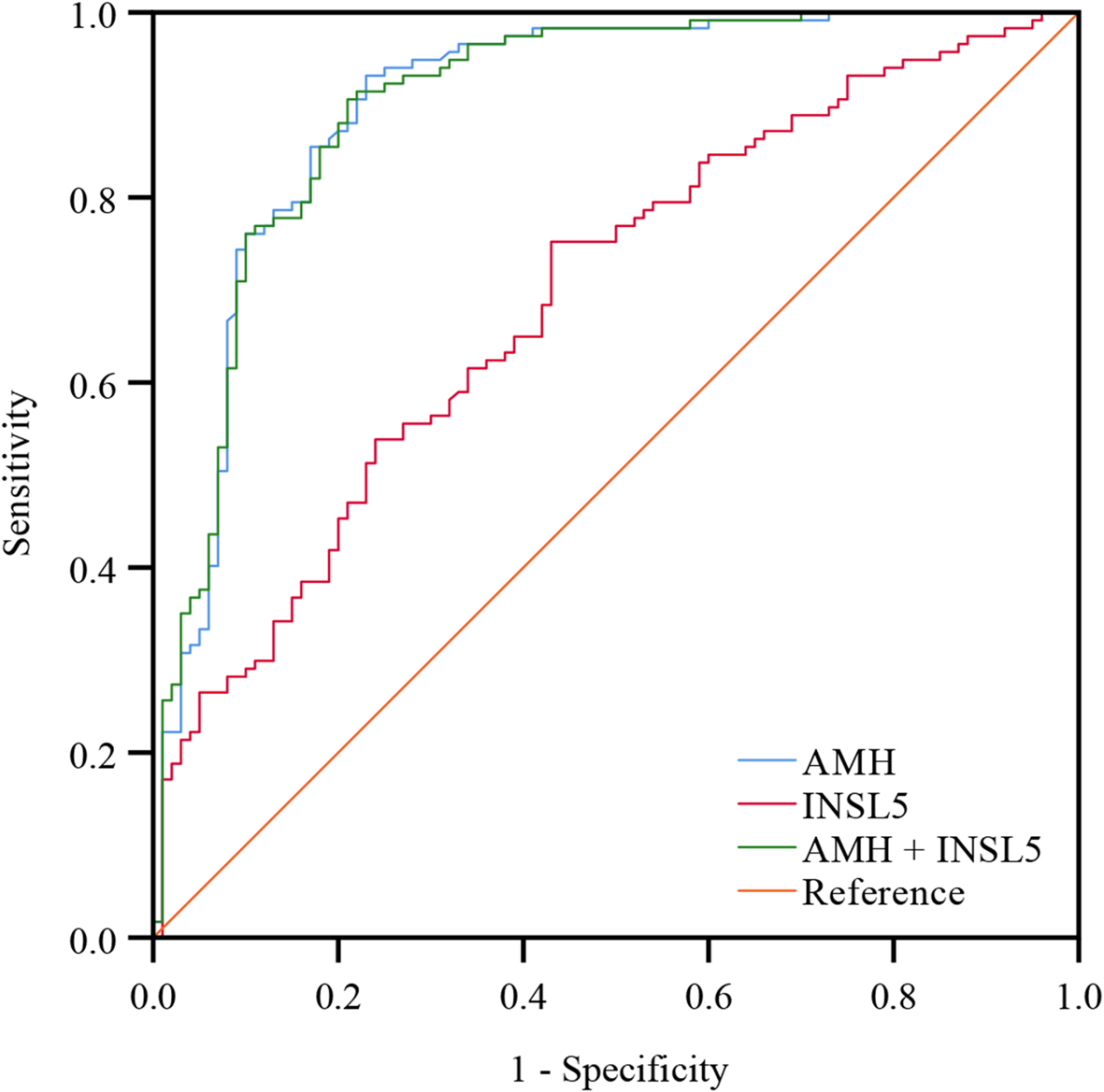
Receiver operating characteristics (ROC) curves of AMH level in INSIL5. The sensitivity (y axis) is plotted against the specificity (x axis)

Women with PCOS had significantly higher INSL5 and AMH levels (Fig. 1). The sensitivity and specificity of the serum concentration of INSL5 and AMH were evaluated for the PCOS group by using cut-off values according to the ROC (Fig. 2, Table 3). The AUC of AMH for the diagnosis value of PCOS was 0.902 (95% confidence interval: 0.858–0.945, *P* < 0.001). The sensitivity for PCOS, with a cut-off value for AMH of 4.56 ng/ml, was 93.2% and the specificity was 77.0%. The AUC of INSL5 for the diagnosis value of PCOS was 0.693 (95% confidence interval:0.623–0.762, *P* < 0.001). When using a cut-off value for INSL5 of 19.88 ng/ml, the sensitivity was 75.2% and the specificity was 57.0%. While the combination of AMH and INSL5 cut-offs did not significantly improve the accuracy of diagnosis value.

**Table 3:**
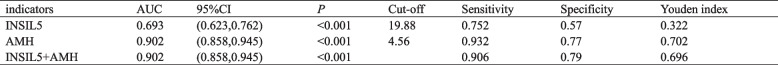
Diagnosis value of INSIL5 and AMH for PCOS

## Discussion

In the present study, we investigated the circulating peptide INSL5 levels in women with PCOS and ascertained relationship between INSL5 and AMH. There are few studies about INSL5, although a previous cross-sectional study suggests that the levels of INSL5 are higher in women with PCOS compared to the normal subjects, consistent with our findings [28]. To the best of our knowledge, this is the first study evaluating the association between INSL5 levels and AMH in women PCOS. Our results showed that women with PCOS had elevated serum AMH levels in the second, third and fourth INSL5 level quartiles compared with the overall sample (Table 1). Linear regression analysis revealed that increased INSL5 levels were associated with higher odds of AMH. The results demonstrated that INSL5 level was positively correlated with AMH in women with PCOS. The origin of follicular overgrowth may be the result of primordial ovarian hyperandrogenism in PCOS, mainly caused by thecal cell dysfunction, but its origin is still unclear [7]. AMH, a recognised biomarker of female reproductive potential, is widely used in clinical practice and is a good indicator of female fertility and menopausal health management. The level of AMH reflects the response and number of primary follicles and response to exogenous gonadotrophins [30].

More studies have shown that the offspring of women with PCOS are at increased risk for future metabolic and reproductive dysfunction [31]. The second follicle abnormality in PCOS is follicular accumulation caused by a dominant follicle selection defect or follicular block. Anovulatory infertility in patient with PCOS is the focus concerns. The change of oocyte capacity and decreased endometrial function are considered as a potential pathogenic factor of fertility in women with PCOS [32, 33]. This process in women with PCOS is primarily associated with impaired FSH, which inhibits follicular maturation and also associated with excessive LH and insulin secretion. Although the underlying mechanism of this process remains unclear, the evidence suggests that higher levels of AMH produced in the polycystic ovary may play a negative selection role in follicular development and inhibit FSH-dependent aromatase activity [19, 34].

INSL5, interacting with RXFP4, is widely distributed among different tissues such as colorectal tissues, placenta, thyroid, thymus, kidneys, ovaries and testis [35]. There is some evidence of a link between INSL5 and the reproductive system in both sexes. RXFP4 expression in the testes of INSL5-deficient mice resulted in infertility due to irregular length of the oestrous cycle and spermatogenesis disorders [23]. Another study demonstrated that the expression of RXFP4 and INSL5 in human sperm inhibited the natural decline of sperm motility, which may be related to a decrease in mitochondrial ROS production [29]. The expression of INSL5 in the mouse hypothalamus and testis indicates that INSL5 is influential on the hypothalamic pituitary-gonadal axis (HPG) and has important effects on reproduction [22].

There are few studies on INSL5 and the reproductive system, but many studies have shown that INSL3, another well-known member of the relaxin superfamily, may affect the reproductive function in males and females. Serum LH levels and the number of ovarian follicles determined by ultrasound have been associated with INSL3 levels in women with PCOS [36]. Future clinical may find that the significant correlation between INSL3 and AMH found in this research may be associated with impaired follicular development in women with PCOS [24]. In addition to this, animal studies have already shown that INSL3 plays a vital role in ovarian follicular development [27]. A previous study demonstrated that the impaired fertility in INSL3 knockout mice is related to prolonged oestrus cycle and accelerated follicular atresia [25]. Based on these data, we suggest a possible interaction between the INSL family and ovarian function. On the other hand, these results might indicate that increased INSL5 up-regulate AMH, although the receptor for INSL5 in granulosa cells has not yet been described.

Moreover, an optimal cut-of value of INSL5 for predicting PCOS was found to be ≥19.88 ng/ml (sensitivity 75.2%, specificity 57%) and an optimal cut-of value of AMH was 4.56 ng/ml (sensitivity 93.2%, specificity 77%). In the present study, the results suggest that the diagnosis value of AMH for PCOS is better than that of INSL5. Meanwhile, the combination of INSL5 and AMH did not improve the accuracy of the test and the diagnosis value.

We confirmed that increased INSL5 concentrations are positively correlated with T concentrations in women with PCOS, which also supports the potential role of INSL5 in follicular membrane cell steroidogenesis. Several studies have shown that androgens, whether from the ovary, adrenal glands or exogenous sources, may promote the growth of ovarian follicles through androgen receptors and reduction of granulosa cell apoptosis [37, 38]. Among women with PCOS, the highest level of INSL3 is described as functional ovarian hyperandrogenism, which is characterized by more severe hyperandrogenism and insulin resistance [39]. Another study showed that a positive correlation was between INSL3, LH, androgen levels and ovarian follicular number [36]. An inverse relationship between circulating INSL5 and T levels in 14 obese men has also been found; where levels of T increased but INSL5 decreased after obese men underwent bariatric surgery [40].

The physiological role of INSL5 in the human reproductive system is still unclear although many studies have reported the effects of INSL5 on lipid and glucose metabolism. We found no significant correlation between INSL5 levels and HOMA-IR (*P* = 0.498), or with fasting insulin and glucose, which does not support some previous studies [28]. Increasing INSL5 has been shown to make no significant difference on food intake, body weight, or glucose control in mice [41]. However, in INSL5 knockout mice, a reduction in the number of beta cells in the pancreas and reduced insulin production was found, which led to a significant increase in blood glucose levels [23]. INSL5 is co-stored with glucagon-like peptide-1 (GLP-1) and peptide YY, and it stimulated insulin, GLP-1 in both vitro and vivo studies [42, 43]. In agreement with some but not all studies, INSL5 did not correlate with total cholesterol, triglycerides, HDL and LDL in our study.

There are some limits in our research. One limitation of this study is that we did not have the data on follicle numbers detected by ultrasound, or INSL3 levels. We didn’t elaborate PCOS phenotypes and explore the effect of PCOS phenotypes and PCOS features. Another important drawback is the lack of accepted international standards for AMH detection, so concentration and cut-off points are method dependent.

The research showed a significant relationship between circulating INSL5 and AMH which may be related to the increased secretion of androgens and chronic anovulation of PCOS. This link may explicate the contribution of this peptide in the development of the PCOS disorder. Moreover, The relationship between INSL5 and female reproductive function may guide clinical applications, such as fertility guidance and drug therapy. The mechanisms responsible for increased concentrations of AMH and particularly INSL5 in the blood of women with PCOS are, however, still unclear and need to be further elucidated in future research.

## Data Availability

All data generated or analyzed during this study are included in the manuscript.

## Abbreviations

INSL5: Insulin-like peptide 5
PCOS: Polycystic ovary syndrome
AMH: Anti-Müllerian hormone
PCO: Polycystic ovaries
GPCRs: G protein-coupled receptors
RXFP: Relaxin family polypeptide receptor
LH: Luteinizing hormone
FSH: Follicle-stimulating hormone
E2: Estradiol
PRL: Prolactin
P: Progesterone
HDL: High-density lipoprotein
LDL: Low-density lipoprotein
DHEA-S: Dehydroepiandrosterone sulfate
T: Total testosterone
SHBG: Sex hormone-binding globulin
FAI: Free androgen index
HOMA-IR: Homeostatic model assessment index of insulin resistance
SD: Standard deviation
ANOVA: Analysed using analysis of variance
ROC: Receiver operating characteristic
AUC: Area under the curve
BMI: Body mass index
GLP-1: Glucagon-like peptide-1
